# Supplementary material for: Evidence supported by Mendelian randomization: impact on inflammatory factors in knee osteoarthritis
Source: Front Med (Lausanne). 2024 May 28;11:1382836. doi: 10.3389/fmed.2024.1382836 (PMC11165061; doi:10.3389/fmed.2024.1382836)
Supplement: Supplementary file 1 [file Data_Sheet_1.ZIP › data/GCST90274777_5e6/777.leaveoneout.pdf]

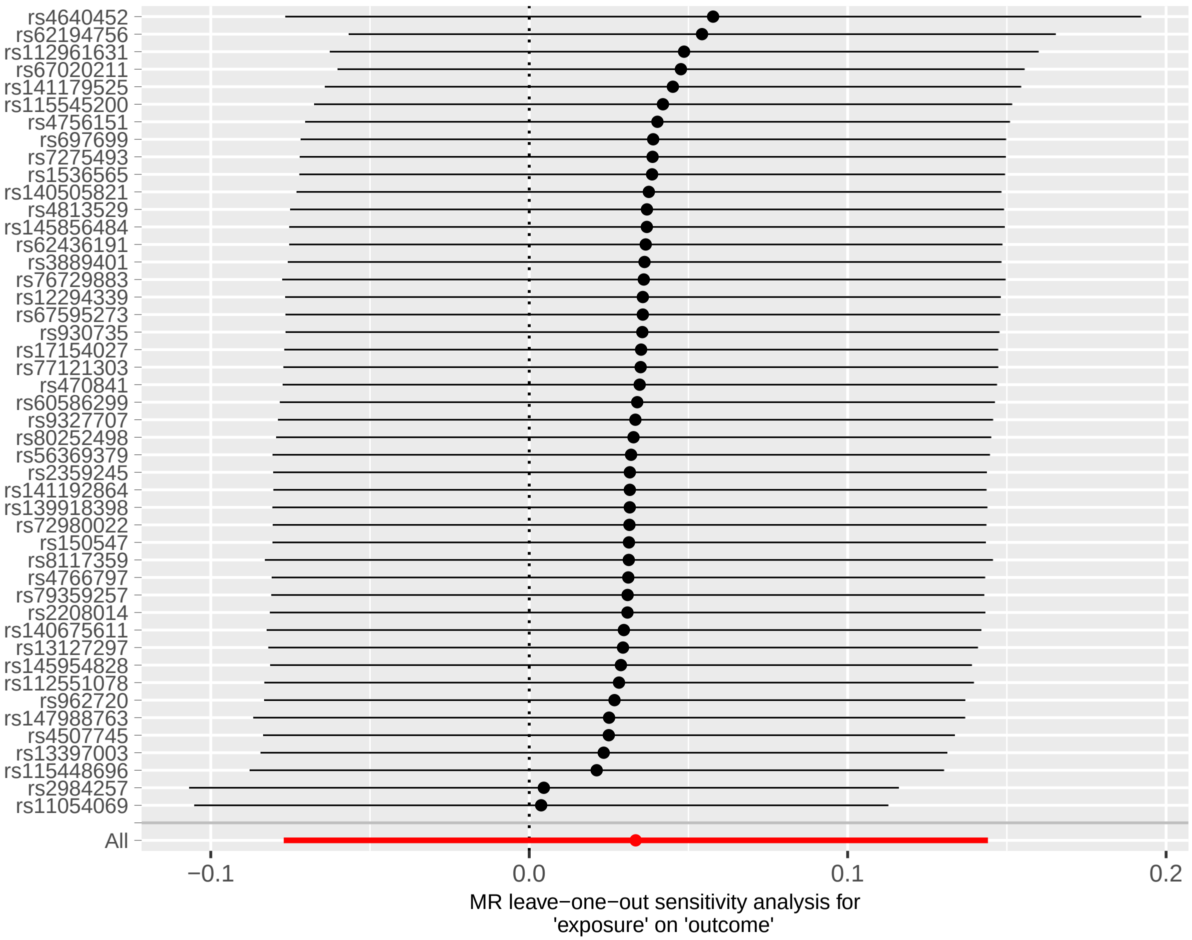
rs4640452
rs62194756
rs112961631
rs67020211
rs141179525
rs115545200
rs4756151
rs697699
rs7275493
rs1536565
rs140505821
rs4813529
rs145856484
rs62436191
rs3889401
rs76729883
rs12294339
rs67595273
rs930735
rs17154027
rs77121303
rs470841
rs60586299
rs9327707
rs80252498
rs56369379
rs2359245
rs141192864
rs139918398
rs72980022
rs150547
rs8117359
rs4766797
rs79359257
rs2208014
rs140675611
rs13127297
rs145954828
rs112551078
rs962720
rs147988763
rs4507745
rs13397003
rs115448696
rs2984257
rs11054069
All
−0.1
0.0
0.1
0.2
MR leave−one−out sensitivity analysis for
'exposure' on 'outcome'
